# The effects of interfacial potential on antimicrobial propensity of ZnO nanoparticle

**DOI:** 10.1038/srep09578

**Published:** 2015-04-15

**Authors:** Manoranjan Arakha, Mohammed Saleem, Bairagi C. Mallick, Suman Jha

**Affiliations:** 1 Department of Life Science, National Institute of Technology Rourkela, Odisha 769008, India; 2 Department of Chemistry, Ravenshaw University, Odisha 753003, India

## Abstract

The work investigates the role of interfacial potential in defining antimicrobial propensity of ZnO nanoparticle (ZnONP) against different Gram positive and Gram negative bacteria. ZnONPs with positive and negative surface potential are tested against different bacteria with varying surface potentials, ranging −14.7 to −23.6 mV. Chemically synthesized ZnONPs with positive surface potential show very high antimicrobial propensity with minimum inhibitory concentration of 50 and 100 μg/mL for Gram negative and positive bacterium, respectively. On other hand, ZnONPs of the same size but with negative surface potential show insignificant antimicrobial propensity against the studied bacteria. Unlike the positively charged nanoparticles, neither Zn^2+^ ion nor negatively charged ZnONP shows any significant inhibition in growth or morphology of the bacterium. Potential neutralization and colony forming unit studies together proved adverse effect of the resultant nano-bacterial interfacial potential on bacterial viability. Thus, ZnONP with positive surface potential upon interaction with negative surface potential of bacterial membrane enhances production of the reactive oxygen species and exerts mechanical stress on the membrane, resulting in the membrane depolarization. Our results show that the antimicrobial propensity of metal oxide nanoparticle mainly depends upon the interfacial potential, the potential resulting upon interaction of nanoparticle surface with bacterial membrane.

Due to rapid growth of nanotechnology, the engineered nanoparticles (NPs) are being widely used in different fields of biomedical and pharmaceutical sciences, like biosensing, antibiotics, imaging, and drug delivery^1^,^2^. Inside the biological medium, NPs interact with cells, membrane, proteins and DNA establishing nano-bio interface, and the functional aspects of the interface depend on colloidal forces as well as physico-chemical interactions^1^. The interaction pattern is based on physico-chemical properties of the interface, for example NP surface potential induces an electrostatic field around it, which in turn reorient local water population up to a certain depth into the bulk, depending upon the electrostatic field strength^3^. The reoriented local water population has potential to rearrange the whole biological mechanisms like protein folding, membrane dynamics, enzyme catalysis etc^3^. Additionally, the interaction at nano-bio interface defines dispersity and compatibility of NPs in the media (inside or outside of cell)^4^,^5^,^6^. Unlike water, inside the cell or biological fluid, interaction of NPs is not only limited to the electrostatic interaction, but other interactive forces like van der Waal's, hydrophobic, hydrophilic forces etc also play important role. The interfacial potential is a result of all these forces present between interacting nanoparticle and biomolecule surfaces. Thus, interfacial potential formed on interaction of NP with biomolecular surfaces becomes very important factor to study, prior to its use for any biological applications. In face of vast applications of nanomaterials in biotechnology and life sciences, antimicrobial and cytotoxic property of nanomaterial has drawn significant interest^7^.

Excess uses of antibiotics and chemical bactericides have resulted in development of resistant bacterial strains, which in turn creates the onset of infectious diseases^8^. To avoid such resistance and the need to develop any resistant strain, researchers are looking for alternatives that can be used as a broad range antimicrobial agent such as NP formulations as an effective antimicrobial agents^9^,^10^,^11^. Metallic NPs with photocatalytic property result in inhibition of microbial growth non-specifically as a result of reactive oxygen species (ROS) generation upon the NPs contact with radiation or media^12^,^13^. The energy band gap of the NPs is so small that on absorption of the radiation, excited electrons of the NPs start cascade reactions for ROS production. However, sensitivity of microbes to these metallic NPs varies according to the interface provided by the bacterial membrane. Gram positive bacteria are found less sensitive to the NPs with respect to Gram negative bacteria because of the presence of a thicker peptidoglycan layer^14^. At the same time, another study showed that the interaction between metallic NP and culture media results in peroxide generation, which is a cause of antimicrobial propensity^15^.

In order to understand the role of interfacial potential on antimicrobial propensity, we investigated antimicrobial propensity of ZnONPs having positive and negative surface potentials against three randomly chosen Gram positive and Gram negative bacteria. To this end, ZnONP interface with positive surface potential showed significant antimicrobial tendency against Gram negative and Gram positive bacteria in comparison to interfaces provided by ZnONP with negative surface potential. We propose that in order to bring significant changes in microbial viability, the interface needs to develop such a potential which results in either physical rupture of membrane (membrane depolarization) or enhanced ROS production (at the interface or inside the bacteria). Although, the antibacterial activity of ZnONP against different bacterial strains is well established^7^,^16^,^17^, however, to best of our knowledge, the role of interaction profile at the interface in antimicrobial propensity of ZnONP has not been reported till date.

## Results

**Figure 1:**
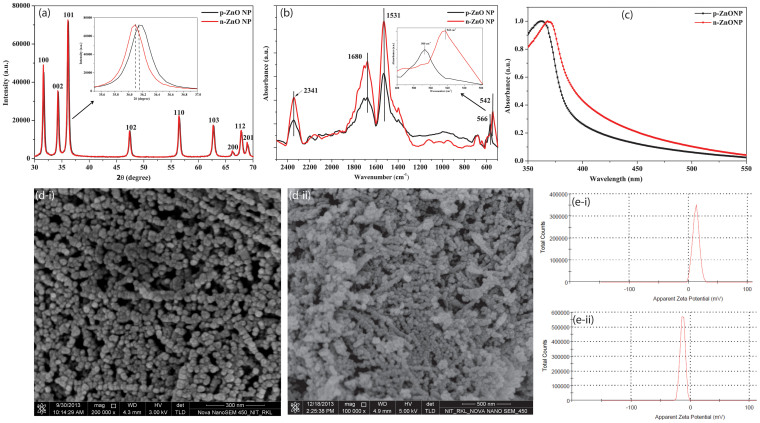
Characterization of ZnONPs. (a) XRD, (b) ATR-FTIR absorption spectra, (c) UV-Vis absorption spectra of p-ZnONP and n-ZnONP, (d) FE-SEM image of p-ZnONP (d-i) and n-ZnONP (d-ii), (e) Zeta potential analysis of p-ZnONP and n-ZnONP showing value of +12.9 mV (e-i) & -12.9 mV (e-ii), respectively.

Initially, ZnO nanoparticle with positive surface potential (p-ZnONP) was synthesized, and the surface was modified to negative surface potential ZnONP (n-ZnONP) using sodium citrate. The X-ray diffraction (XRD) data (Fig. 1a) of p-ZnONP and n-ZnONP revealed that both the samples prepared are crystalline in nature with peaks at different 2θ, i.e. angle values 31, 34, 36, 47, 56, 62, 66, 67 and 68 corresponding to different indices (100), (002), (101), (102), (110), (103), (200), (112) and (201), respectively. The indices are well indexed to the hexagonal wurtzite structure of bulk ZnO lattice parameters, as suggested by different studies^18^,^19^,^20^. Additionally, the analysis of XRD spectra of p-ZnONP and n-ZnONP using X′ pert high score software with search and match facility demonstrates that both types of synthesized NPs have hexagonal ZnO crystals (JCPDS reference code–80-0074 and 79-0208 for p-ZnONP and n-ZnONP, respectively). Interestingly, it is observed in the XRD spectra that the diffraction peaks of n-ZnONP are slightly shifted towards the lower Bragg angle compared to p-ZnONP (inset of Fig. 1a). The shifting of peaks reveals the lattice expansion upon sodium citrate coating leading into increased interlayer spacing of n-ZnONP along the c-axis^21^. The average particle size for p-ZnONP and n-ZnONP are determined using Scherrer's equation
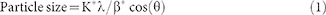
Where λ is the wavelength of X-ray (1.540 × 10^−10^ m), K = 0.9, proportionality coefficient (shape factor), θ is the Braggs angle, and β is the full width at half maximum in radians. On applying equation(1), particle size of ZnONPs are calculated to be 30 and 39 nm for p-ZnONP and n-ZnONP, respectively. The theoretical specific surface area (SA) of synthesized nanoparticles are also determined using the equation SA = 6/(D*ρ), as suggested by Hjiri et al., where D represents the particle size, ρ represents the theoretical density of ZnO (5.606 g/cm^3^)^21^. Using the equation, the theoretical specific surface area of p-ZnONP and n-ZnONP are found to be 35.67 and 27.44 m^2^/g, inferring that specific surface area decreases upon surface coating.

The compositions of p-ZnONP and n-ZnONP were analyzed using Attenuated Total Reflection-Fourier Transform Infrared (ATR-FTIR) spectroscope, shown in Fig. 1b. Strong absorption peaks at 1531 cm^−1^ and 2341 cm^−1^, for both types of NPs are due to bending vibrations of N-H and the asymmetric stretching vibration of C = O bonds, respectively. The absorption peaks observed at 1680 cm^−1^ are due to vibration of C = O bond present in the residual acetate/carbonate or citrate formed in the process. The absorption peaks below 800 cm^−1^ provides important information about internal metal-oxygen bond vibration^22^. The spectra of materials showed absorption peak near 542 and 566 cm^−1^ for n-ZnONP and p-ZnONP respectively, corresponding to Zn-O bond stretching vibration present in nanocrystal lattice (Fig. 1b inset). The shift in peak from 566 to 542 cm^−1^ for Zn-O-Zn bond interprets that p-ZnONP required higher frequency vibration to vibrate Zn-O-Zn bond compared to the bond present in n-ZnONP; frequency of vibration is inversely proportional to square root of the mass of the vibrating molecule, *Hooke's Law*. Thus, the presence of citrate as coating on the surface of ZnONP was resulting into lower wavenumber vibration for Zn-O-Zn bond present in n-ZnONP compared to p-ZnONP. Additionally, the modification is further confirmed at bond level using ATR-FTIR spectroscopy, where peak intensities corresponding to C = O vibrations, i.e. 2341 and 1680 cm^−1^ are found to be enhanced for n-ZnONP compared to p-ZnONP.

Surface plamon resonance (SPR) is a characteristic property of NPs, especially photocatalytic metal NPs with short band gap, an energy gap between top vibrational level of valence band and bottom vibrational level of conduction band. Figure 1(c) shows the UV-Vis absorption spectra of p-ZnONP and n-ZnONP with absorption peaks at 362 and 369 nm respectively, which are attributed to SPR property of ZnONPs^20^,^23^,^24^,^25^. The absorption peak at 362 nm obtained for p-ZnONP is very close to the absorption peak of 364 nm as obtained by Tankhiwale et al.^23^ and 361 nm by Vigneshwaran et al.^24^. From the figure, it is evident that upon coating of sodium citrate the absorption peak for ZnONP shifted from 362 nm to 369 nm, i.e. red shift, confirming the surface modification of ZnONP. The shifting of absorption peak towards higher wavelength side is due to decrease in band gap of NP, which is due to increase in particle size. The energy levels in nanomaterials are discretely defined and the shifting of energy levels obeys the quantum size effect. The energy levels become indiscrete with increasing size of the nanomaterials, due to which the band gap decreases^26^. The band gap energy (E_bg_) of synthesized ZnONPs is determined using the equation, E_bg_ = 1240/λ (eV)^20^, where E_bg_ and λ represent for band gap energy in eV and wavelength in nanometer, respectively. The band gap energy of p-ZnONP and n-ZnONP are 3.4 and 3.3 eV respectively, which are very close to the theoretical values of ZnONP, as supported by different literatures^20^,^27^,^28^.

Field Emission Scanning Electron Microscopy (FE-SEM) images of both positive and negative potential ZnONPs suggest that the particles are spherical in shape with diameter range of 25–35 nm (Fig. 1d-i) and 35–45 nm (Fig. 1d-ii), respectively. The increase in size of negative potential ZnONP confirms the coating. Zeta potential measurement shows that the synthesized p-ZnONP has surface potential of +12.9 mV, while surface modification with citrate provides surface potential of −12.9 mV (n-ZnONP) (Fig. 1e-i &-ii).

### ZnONP-Bacteria interfacial potential

**Figure 2:**
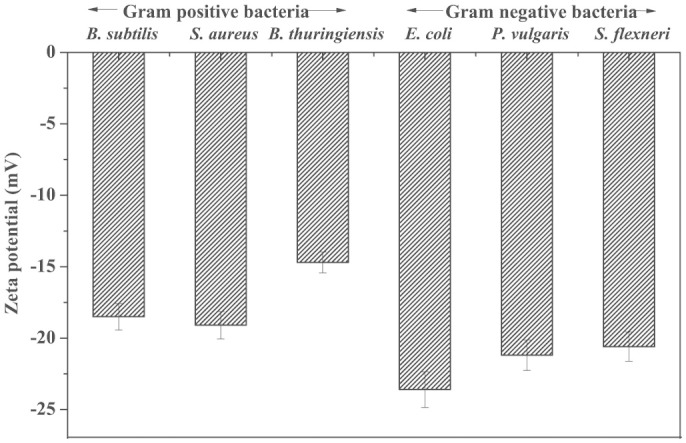
Zeta potentials of Gram positive and Gram negative bacteria.

To study the effect of interfacial potential on antimicrobial propensity of ZnONPs, different bacteria with varying surface potential are used in the study. Fig. 2 shows the zeta potential value of both Gram positive and Gram negative bacteria used in the study. Negative zeta potential values are obtained for all tested organisms. However, Gram negative bacteria exhibited higher negative potential than Gram positive bacteria, due to presence of additional layer of negatively charged lipopolysaccharide (LPS) compared to Gram positive bacteria.

**Figure 3:**
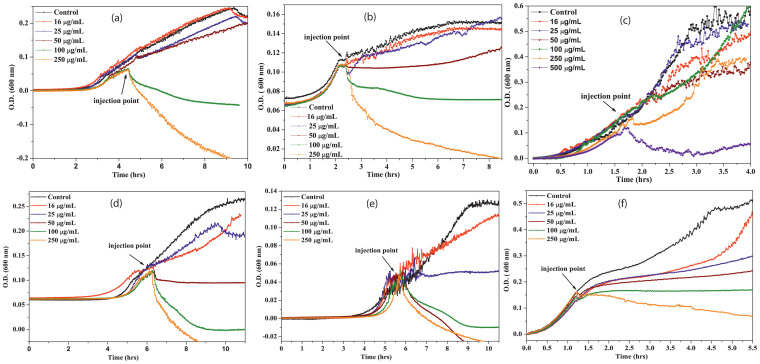
Growth kinetics of bacteria (a. *B. subtilis*, b. *S. aureus*, c. *B. thuringiensis*, d. *E. coli*, e. *S. flexneri*, and f. *P. vulgaris*) in presence of different concentrations of p-ZnONPs. In each case, black line shows the growth kinetic curve of untreated cells. Different concentrations of p-ZnONP taken were 16, 25, 50, 100, 250, and 500 (only for *B. thuringiensis*) μg/mL, and injected at the mid log phase of growth kinetics, as shown by arrow.

The growth kinetic studies are carried out in presence and absence of p-ZnONP and n-ZnONP in order to observe the minimum inhibitory concentration (MIC), as shown in Fig. 3. From the figure, it is evident that lower concentrations of p-ZnONP (i.e, 16, 25 and 50 μg/mL) do not show significant effect on growth kinetics of the Gram positive bacteria (Fig. 3a–c). However, 100 μg/mL and higher concentrations of p-ZnONP in culture exerts significant growth inhibition for *Bacillus subtilis* and *Staphylococcus aureus*, while *Bacillus thuringiensis* shows relatively higher resistance against positively charged p-ZnONP. The bacteria only shows significant growth inhibition above 500 μg/mL of p-ZnONP. Although, 250 μg/mL of p-ZnONP delays the growth of *B. thuringiensis*. However, upon adoption to the stress condition, the bacteria re-starts the growth after a short dormant phase. Hence, the complete inhibition of *B. thuringiensis* growth kinetic happens above 500 μg/mL of p-ZnONP. Additionally, the Fig. 3d–f represent the effect of varying concentrations of p-ZnONP on the growth kinetics of Gram negative bacteria. The growth curves for *Escherichia coli* and *Proteus vulgaris* only show the inhibition above 50 μg/mL of p-ZnONP. However, in case of *Shigella flexneri*, inhibition of bacterial growth starts from concentration of 25 μg/mL p-ZnONP.

**Figure 4:**
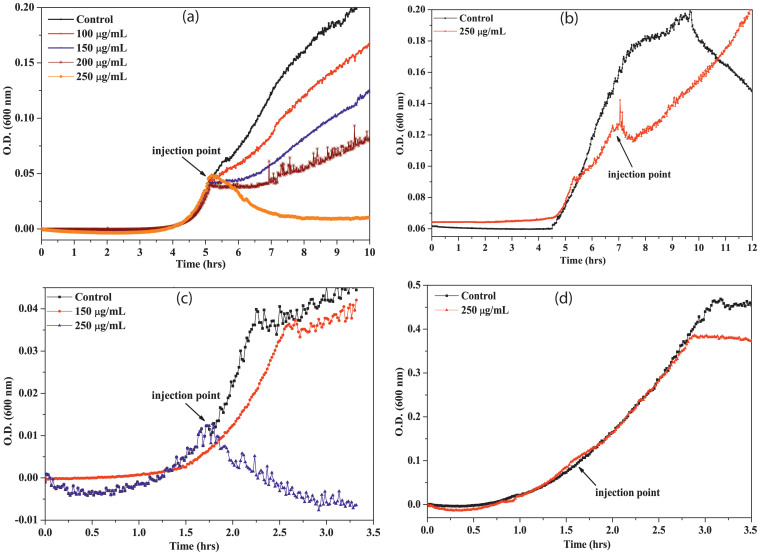
Growth kinetics of bacteria in the presence of different concentrations of n-ZnONP. In each case, black line shows the growth kinetic curve of untreated cells. Both Gram positive (a. *B. subtilis*) and Gram negative (b. *E. coli*, c. *S. flexneri*, d. *P. vulgaris*) bacteria were treated up to 250 μg/mL of n-ZnONP (injected at the mid log phase of growth kinetics, as shown by arrow).

Figure 4(a) shows the growth kinetics of *B. subtilis* in presence of n-ZnONP. From the figure, it is evident that the bacteria shows normal growth in presence of n-ZnONP concentrations below 200 μg/mL, but the inhibition of bacterial growth occurs at 250 μg/mL. The value is much greater than the concentration of p-ZnONP (100 μg/mL) needed to completely suppress the growth of bacteria. Figure 4b–d show the growth kinetics of Gram negative bacteria in presence of n-ZnONP. Like Gram positive bacteria, the growth of *E. coli* and *P. vulgaris* are also unaffected in the studied range of n-ZnONP concentrations. However, 250 μg/mL of n-ZnONP completely inhibit *S. flexneri* growth. Nevertheless, the inhibition concentration is much higher than those observed for p-ZnONP against the bacterium.

**Figure 5:**
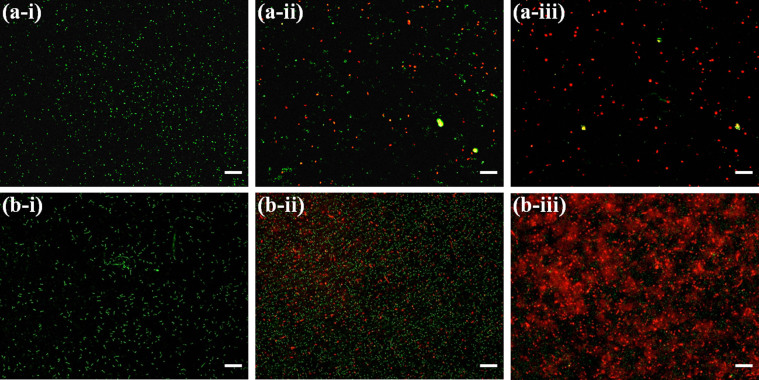
Fluorescence microscopic images of the green and red fluorescence stained *B. subtilis* and *E. coli* in absence and presence of p-ZnONP; *B. subtilis* (a-i), *B. subtilis* in presence of 100 μg/mL of p-ZnONP (a-ii), and 250 μg/mL of p-ZnONP (a-iii), *E. coli* (b-i), *E. coli* in presence of 50 μg/mL of p-ZnONP (b-ii), and 250 μg/mL of p-ZnONP (b-iii). The scale bars represent for 20 μm.

Moreover, LIVE/DEAD BacLight Bacterial Viability fluorescence Kit is used to distinguish the non-viable cells from viable cells, which resulted from disintegration of the membrane upon the nanoparticle treatment. According to the principle and as shown in the Fig. 5, viable bacterial cells having intact cell membrane are stained green by the Syto9 fluorescence dye, whereas non-viable bacterial cells with deformed cell membrane upon NP treatment are stained red by propidium Iodide fluorescence dye^29^. As shown in figure 5a-i & b-i, untreated *B. subtilis* and *E. coli* cells exhibited green fluorescence indicating presence of 100% viable bacterial cells, whereas the p-ZnONP treated bacterial samples show a mixture of red and green fluorescence confirming a mixture of viable and non-viable cells (Fig. 5a-ii & b-ii). In presence of 250 μg/mL of p-ZnONP, the fraction of bacterial cells exhibiting red fluorescence is upto 90%, indicating loss of the membrane integrity and cell viability (Fig. 5a-iii & b-iii). However, in presence of 250 μg/mL of n-ZnONP, the fraction of red fluorescent *E. coli* cell is insignificant (data not shown) compared to the untreated cells.

**Figure 6:**
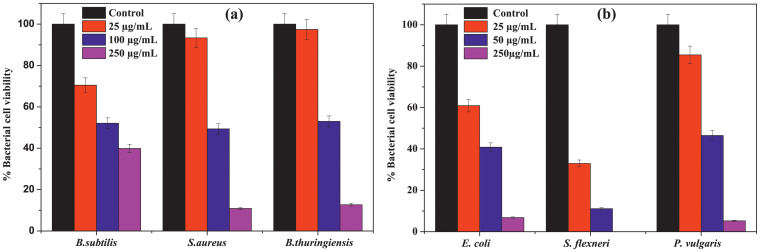
Quantification of bacterial cell viability at different concentrations of p-ZnONP. Colony forming units (CFU) were quantified for both Gram positive and Gram negative bacteria, and expressed as percentage of viable cells.

The cell viability of both Gram positive and Gram negative bacterium in presence of different concentrations of p-ZnONP is further supported by the colony forming unit (CFU) study, as shown in Fig. 6. The CFU results are in accordance with growth kinetic study for both Gram positive and Gram negative bacteria, as well as the BacLight fluorescence microscopic study. The MIC of p-ZnONP for both types of bacteria is evaluated from CFU measurement, shown in Table 1. The reduction in number of viable cells with increasing concentration of ZnONP confirms the antibacterial activity of ZnONP towards selected bacteria.

### Surface potential neutralization of *B. subtilis* and *E. coli* by ZnONPs

**Figure 7:**
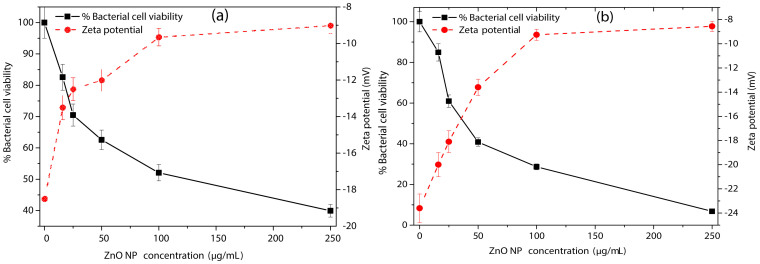
Effect of p-ZnONP on bacterial cell viability and surface zeta potential of *B. subtilis* and *E. coli* cells. *B. subtilis* (a) and *E. coli* (b) cells were treated with increasing concentrations of p-ZnONP like 16, 25, 50, 100, 250 μg/mL. Solid black lines represent the relative percentage of viable bacterial cells, whereas dashed red lines correspond to zeta potential values at different concentrations of p-ZnONP. Triplicate experiments were done for each reactions, and error bar represents the standard error of mean.

Zeta potential measurements are carried out to examine the effects of ZnONPs on the membrane surface potential. As shown in Fig. 7, in absence of NPs, *B. subtilis* and *E. coli* cells display zeta potential of −18.5 mV and −23.6 mV, respectively. However, the potential moved to neutral as increasing concentrations of p-ZnONP are added. On the other hand, addition of increasing concentrations of n-ZnONP show insignificant change in interfacial potential for both the bacterium. The observation indicates insignificant potential neutralization upon n-ZnONP addition. Although, the interfacial potentials at highest studied concentration of p-ZnONP for both the bacterium are not same, but change in magnitude of interfacial potentials are capable of destabilizing the respective bacterial membrane via enhanced ROS production and/or surface tension. Both the factors are explored in next section using the fluorescent dye, 2', 7'-Dichlorodihydrofluorescein diacetate (DCFH-DA), and the SEM/FE-SEM for high resolution images for any possible membrane deformities.

The surface neutralization study is conducted in HEPES (4-(2-hydroxyethyl)-1-piperazineethanesulfonic acid) buffer following wash of bacterial cells with HEPES buffer to eliminate the presence of different molecular surfaces arising from nutrients present in Muller Hinton Broth (MHB). To prove the effect, we conducted the experiment in MHB medium instead of HEPES buffer (supplementary Fig. S5), and the data show insignificant change in surface potential upon nanoparticle treatment. Habash et al.^30^ and Domingues et al.^31^ have also observed same effect of nutrient broth and salt on zeta potential values, respectively.

### Enhanced ROS production in presence of ZnONP-bacteria interface

**Figure 8:**
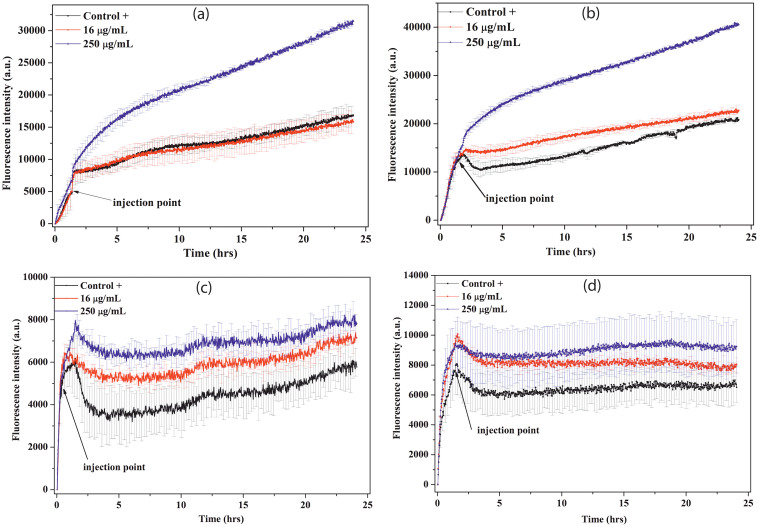
ZnONPs induced ROS detection. *B. subtilis* cells (figure a and c) and *E. coli* cells (figure b and d) were treated with 16 μg/mL (red curve) and 250 μg/mL (blue curve) of positively charged (panel a and b) and negatively charged (panel c and d) ZnONPs, and ROS were detected by measuring fluorescence emission intensity at 523 nm. In each case, except control, NPs were added in the log phase of bacterial growth. The fluorescence emission intensity are compared with positive control (without injection of NPs, black curve) in each case. Each curve represents the average of three independent measurements with corresponding standard error of mean.

The surface potential neutralization of bacteria triggers the production of ROS, which is considered responsible for lipid, protein and DNA damage, resulting into non-viable bacterial population^32^,^33^. Change in ROS production upon addition of ZnONP has been evaluated using the fluorescence dye, DCFH-DA. DCFH-DA is known as peroxynitrite indicator, which detects both hydrogen peroxide and nitric oxide, and considered as ROS indicator^34^. The dye on oxidation has excitation and emission maxima at 503 and 523 nm, respectively. Thus, in order to study the ROS production, the culture was inoculated with DCFH-DA dye which gets oxidized with production of ROS, and gives the emission at 523 nm on exciation with 503 nm, as shown in Fig. 8. The figure indicates that ROS is produced also in absence of ZnONPs, i.e. in control culture, since the dye is showing increasing quantum yield with bacterial growth (black line, control+). Nevertheless, the produced ROS in non-stress condition is counteracted by ROS scavenging enzymes present in bacteria. However, in presence of 250 μg/mL of NPs, ROS production is relatively very high, increased by 100–200%, exceeding the capacity of ROS scavengers and resulting in reduced population of viable bacterial cells (Fig. 8). On comparing Fig. 8a&c with Fig. 8b&d respectively, it is evident that production of ROS is relatively higher in presence of p-ZnONP than n-ZnONP for the species, *B. subtilis* and *E. coli*. Additionally, *E. coli* culture shows higher ROS production on p-ZnONP treatment in comparison to *B. subtilis* culture, which can be rationalised with the difference in magnitude of change in interfacial potential for both the bacterium (Fig. 7a & b). Thus, the data, besides supporting observations from the kinetic studies, CFU, BacLight fluorescence measurements, and potential neutralization studies, indicate that the production of ROS on interaction of ZnONP with bacterial membrane mainly result in non-viability of bacterial populations.

### Surface morphology of bacteria upon ZnONP treatment

**Figure 9:**
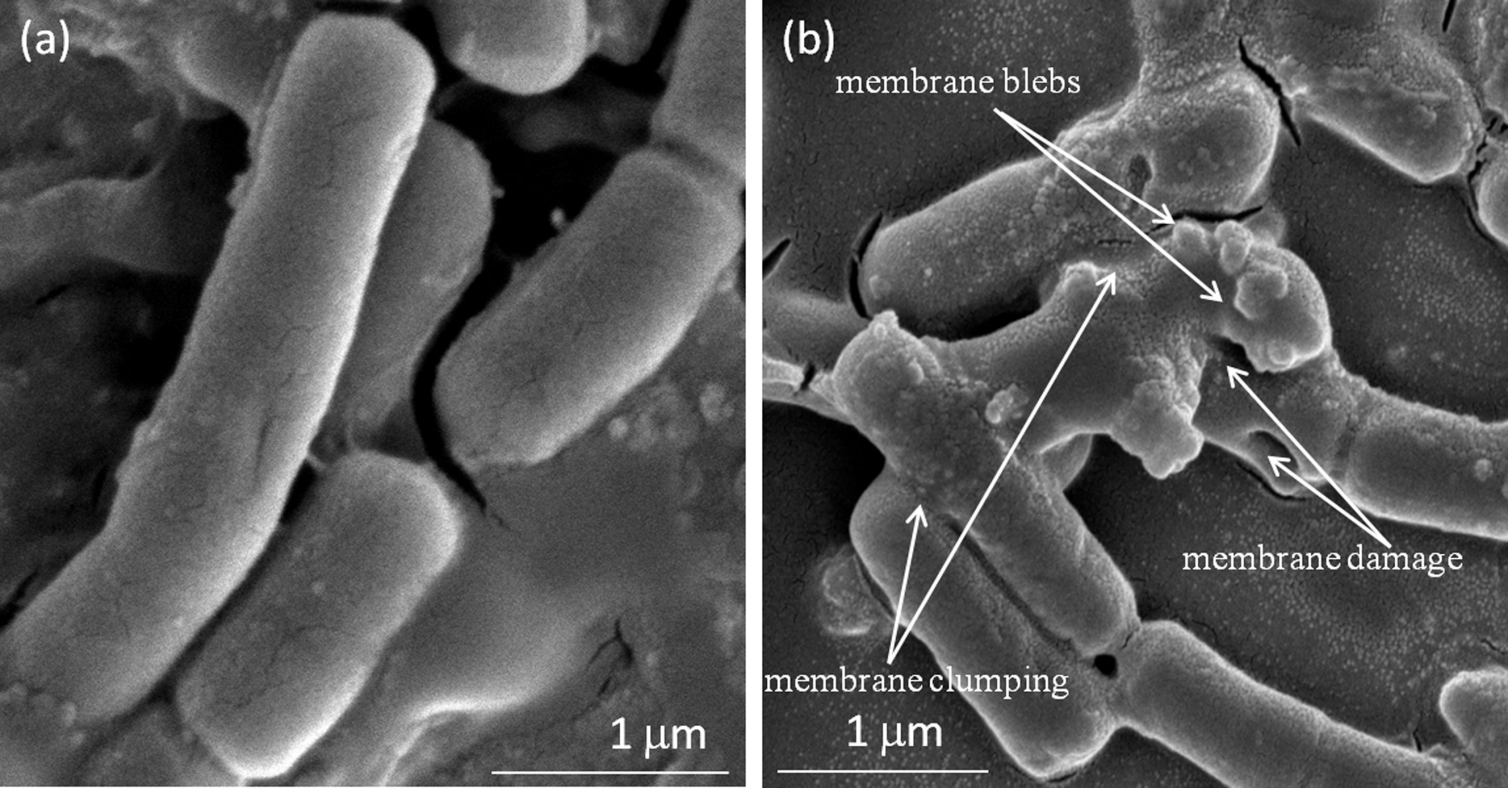
Visualization of ZnONP treated *E. coli* cell surface by FE-SEM, (a) control (without ZnONP treated cells), (b) showing membrane blebbings, membrane damage, and membrane clumping in ZnONP treated cells.

To observe the membrane deformities upon the NPs treatment, the phase contrast, scanning electron microscope (SEM) and FE-SEM were used. The images, obtained using the phase contrast microscopy, show the clumping or aggregation of cells (Supplementary Fig. S1 and S2). For further details, we scanned the NPs treated and untreated bacterial cells using SEM. The images indicate more clumping and membrane rupture in treated cells than the untreated cells (Supplementary Fig. S3 and S4). Additionally, images obtained using FE-SEM helped in detail investigation of topological changes in bacterial membrane (Fig. 9). Upon interaction with p-ZnONP, the bacterial membrane surface potential is neutralized, resulting into increase in surface tension. Above certain p-ZnONP concentration, the interactions result in surface tension change which lead into the membrane depolarization at the point of contact. As a result, bacterial membrane show abnormal textures like membrane rupture, membrane blebs, in images obtained using FE-SEM (Fig. 9b). The ruptured cells no longer remain intact, often found in aggregates or clumps (Fig. 9b).

## Discussion

Although various biological studies have been done to demonstrate the antimicrobial activity of different NPs against different Gram positive and Gram negative bacteria, still mechanism underlying the concept is a matter of intensive research for safe use of NPs as modern antibiotics. As reported by different literatures, various proposed mechanisms of antimicrobial activity of NPs include the generation of ROS (like hydroxyl radicals, superoxide anions, hydrogen peroxide), release of Zn^2+^ ions, cell wall damage, penetration of the cell envelop etc.^15^,^17^,^35^. The work aimed to explore the mechanism in a new dimension by elucidating the biophysical events happening at the interface of NP and bacteria, leading into various changes resulting into bacterial non-viability. Here, we have taken ZnONP due to its strong antimicrobial activity as reported by different literatures and wide applications in various fields of biological sciences^17^. From the set of experiments, we hypothesized a sequence of events happening at the interface, like (i) resulting interfacial potential lead to attachment of NPs on bacterial membrane, (ii) simultaneous neutralization of bacterial surface potential resulting into electron-hole pair generation in proximity, which (iii) enhances the production of ROS. The sequences, altogether, guide bacteria onto a path which leads into non-viable cells.

Due to additional layer of negatively charged lipopolysaccharides, Gram negative bacteria are more negatively charged than Gram positive bacteria^36^, which were also confirmed from our zeta potential measurement studies for the bacterium (Fig. 2). To prove the first event of the hypothesis, we synthesized two types of ZnONPs having opposite potentials (+12.9 mV and −12.9 mV) and growth kinetic studies have been performed in presence of the NPs. The MIC of p-ZnONP for both Gram positive and Gram negative bacteria varied in range of 50–100 μg/mL, which is further supported by CFU measurement studies. To gain further insights into these interaction events, growth kinetic studies have been performed in presence of the NP with negative surface potential. Since bacterial surface possess negative potential and our modified ZnONP is also having negatively surface potential, there would be a relatively repulsive interaction between the surfaces. The growth kinetic study of *B. subtilis*, which is a Gram positive bacteria with relatively less negative surface potential among the studied bacteria, showed inhibition at 250 μg/mL of n-ZnONP, only. However, the value is two and half fold higher than that found for p-ZnONP against same bacteria, i.e. 100 μg/mL. To investigate more about the interfacial potential between NPs and bacteria surfaces, we have taken three Gram negative bacteria due to their higher negative surface potential than Gram positive bacteria. For all the bacterium, higher MIC was observed for n-ZnONP (Fig. 4b–d) compared to the value found for p-ZnONP (Fig. 3d–f). The observations clearly indicate that the possible interaction between the nanoparticle and bacterial membrane surfaces result in the interfacial potential which triggers possible reactions leading to bacterial non-viability.

The term surface neutralization is largely attributed, in biological system, to balance the surface potential that exist between accessible negatively charged, polar and non-polar functional groups on bacterial surface and the interacting entities present on p-ZnONP surface^36^. Since *E. coli* is Gram negative bacteria, possess more negative surface potential than *B. subtilis*, which is a Gram positive bacteria (Fig. 2). Increasing concentrations of p-ZnONP take the interfacial potential at the p-ZnONP-bacteria interface to neutral, suggesting the neutralization of surfaces by the respective surface functional groups present on the interacting partner. As a result of the neutralization, the energy released is possibly either utilized in the production of ROS or membrane surface tension or both, as indicated in the work of Espita P.J.P. et al.^37^. The work suggested that generation of ROS on the surface of ZnONP play role in the antimicrobial activity by ZnO nanopatricles following the possible reaction steps given below^37^. 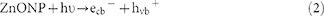

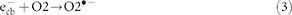

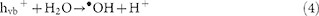

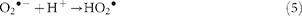

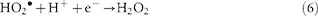


Since ZnONP is a photocatalytic material having a band gap of 3.3 eV^20^. Hence, energy released higher than the band gap energy, can trigger the movement of electrons from the valence band (vb) to the conduction band (cb) resulting a positive area in the valence band (electron hole, h^+^) and free electrons (e^−^) in the conduction band^38^. When ZnONP is in suspension, the created electron-holes react with H_2_O molecules and separate the H_2_O molecules into ^•^OH and H^+^. Simultaneously, dissolved O_2_ molecules in the medium are transferred into O2^•−^ (superoxide anion radicals) and react with H^+^ ions to generate HO_2_^•+^ followed by collision with an electron to produce H_2_O_2_ molecules^37^,^38^,^39^. The reaction occurs at the interface and produces reactive oxygen species, among which hydroxyl radicals and superoxide anion radicals are negatively charged. The charged radicals can not penetrate the cell membrane, since the bacterial cell membrane is negatively charged^40^. However, modification of the membrane physico-chemistry can not be ruled out while the generation of ROS is happening in the proximity. Since H_2_O_2_ is uncharged reactive oxygen species, the molecule can penetrate inside the bacteria and cause the non-viability^40^. The amount of hydrogen peroxide generated is directly proportional to the concentration of p-ZnONP in suspension. The increase in concentration of p-ZnONP increases the number of possible interactions leading into ROS production, and hence antibacterial activity increases^37^.

The DCHF-DA dye is a cell permeant dye, and indicator of reactive oxygen species. The initial ROS formation, i.e. before injection of NP, is due to metabolic activities (Fig. 8), which is approximately same for all cases. Above MIC, the fluorescence intensity upon the nanoparticle addition increased many fold (Fig. 8a, b), supporting the work of Espitia P.J.P. et al. It is very interesting to observe that at 250 μg/mL of p-ZnONP, the emission intensity in *E. coli* culture is higher than the intensity observed in *B. subtilis* culture, inferring production of more ROS leading to more cell death. The observation is similar to our growth kinetics and CFU results for both the bacteria. In case of n-ZnONP, the increased emission intensity of the dye is insignificant compared to control cultures. The observations rationalize the interaction between the negative surface potentials result in interface that can not produce or enhance reactive oxygen species generation. Thus, the observation strongly supports first and second events of the hypothesis.

The effect of the interactions on bacterial cell viability is further explored using the BacLight kit fluorescent microscopic method, which distinguishes viable or non-viable cells based on the membrane integrity. The kit uses mixture of two fluorescent dyes, Syto9 and propidium Iodide (PI) dyes, which stains green (Syto9) to viable cells with intact membrane and stains red (PI) to non-viable cells with ruptured membrane. The images obtained using the BacLight kit indicate loss of membrane integrity or alteration in membrane permeability on p-ZnONP treatment^29^. Hence, the resulting interfacial potential on interaction of the nanoparticle with bacterial membrane also result in membrane rupture either because of ROS or increased surface tension of bacterial membrane. The later case is further investigated using phase contrast microscopic and SEM/FE-SEM through morphological change in membrane of the bacteria. The images obtained using phase contrast microscope reveal the aggregation/clumping of bacterial cells, whereas images from SEM demonstrate membrane rupture along with aggregation/clumping of bacterial cells. Additionally, the high resolution images obtained using FE-SEM, indicate occurring of membrane blebs along with the events. Upon addition and incubation of p-ZnONP with bacterial cells, neutralization of surface potential was observed, as a result of interaction at the interface leading into increased surface tension. The increased surface tension is capable of affecting the bacterial membrane to a great extent. As a result of above events, bacterial membrane show some abnormal textures like rupture, blebs etc. The ruptured cells no longer remain intact and result in aggregates/clumps^41^.

In conclusion, two types of ZnONPs having opposite surface potentials of the same magnitude were synthesized. Based on the data, insights into the biophysical events happening at the interface of ZnONP-bacteria were gained. Firstly the interaction at the ZnONP-bacteria interface was explored, and exploration of this concept guided us for understanding the proper mechanism behind the attachment of NPs to bacterial surface. Secondly, the resultant interfacial potential, measured using zeta potential measurement study and standard antibacterial activity assay, helped us to establish a correlation between the interfacial potential and antimicrobial propensity of the NPs. Together, the bio-nano interfacial potential result in a surface tension generating high lateral stress in the membrane leading to irreversible membrane damage via membrane blebbings or rupture, as clearly visible in images obtained using fluorescence microscope, SEM, and FE-SEM. At the end, the molecular events leading to the antimicrobial activity of ZnONP was explored by evaluating ROS production from the interaction at different concentrations of ZnONPs. Taking altogether, the biophysical and antimicrobial data obtained from the study, we hypothesize that the interfacial potential at the ZnONP-bacteria interface is largely responsible for the antimicrobial propensity of ZnONPs.

## Methods

Zinc acetate dihydrate, urea, glutaraldehyde were purchased from Merck (India). Nutrient broth, Mueller Hinton Broth, nutrient agar, agar-agar, tannic acid used for antimicrobial studies were purchased from HIMEDIA, India. HEPES buffer and sodium citrate used for surface modification of ZnONP were purchased from sigma Aldrich (India). 2',7'-Dichlorodihydrofluorescein diacetate (DCHF-DA) was purchased from Cayman chemicals. All chemicals used for this work were of analytical grade, and used without further purification. Different bacterial strains used for antimicrobial studies, like *Bacillus subtilis* (MTCC 736), *Bacillus thuringiensis* (MTCC 8998), *Staphylococcus aureus* (MTCC 737), *Escherichia coli* (MTCC 443), *Shigella flexneri* (MTCC 1457), *Proteus vulgaris* (MTCC 426), were purchased from Institute of Microbial Technology (IMTECH), Chandigarh, India.

### Synthesis of ZnONPs

Zinc oxide NP was synthesized by chemical precipitation method using zinc acetate dihydrate and urea as described by Bhattacharjee et al. with some modifications^42^. In brief, 0.1 M of each zinc acetate dihydrate and urea solutions were prepared in deionised water, followed by mixing in a beaker maintaining a volumetric ratio of 1:4. The mixture was vigourously stirred at room temperature to get a homogeneous solution, and heated at 115°C in a muffle furnace for 1.5 hrs, maintaining a closed system. As soon as the reaction was completed, a white precipitate was formed. The precipitate was centrifuged at 6000 rpm for 10 mins, pellet was collected and washed three times with deionised water to remove absorbed chemicals or ions, since the chemicals and ions help in agglomeration of NP. The washed pellet was dried at 100°C, followed by calcination in a muffle furnace at 300°C for 2 hrs. The resulting white powder was characterized for positively charged ZnONP.

For negatively charged ZnONP, surface modification of ZnONP prepared earlier was carried out using 1% citrate buffer. 20 grams of ZnONP was suspended in 1% sodium citrate, 10 mM HEPES buffer, and vigorously vortexed for 5 minutes followed by sonication for 10 min. The above solution was centrifuged at 6000 rpm for 30 min, and the pellet was collected and washed twice in deionised water. Thereafter, the pellet was dried in hot air oven to get the negatively charged ZnONP^43^.

### Nano-material characterization

The XRD patterns of both p-ZnONP and n-ZnONP were recorded on an Ultima IV model Rigaku X-ray diffractometer (Tokyo, Japan) using CU-Kα radiation at a scan rate of 20°/min with step size of 0.05 degree over 2θ range of 25 to 70 radians. The X′-pert high score software having search and match facility was employed to study the different phases present in the samples. The morphological features like shape and size of synthesized NPs were studied using FE-SEM (Nova Nano SEM 450, FEI company), whereas the surface plasmon resonance properties of both types of NPs were analyzed using UV-Vis spectrophotometer (Cary 100, Agilent Technology, Singapore) in absorbance mode. The FTIR spectra of both types of NPs were recorded on an alpha platinum attenuated total reflection (ATR)-FTIR spectrophotometer (Bruker, Germany). The spectra were obtained in ATR mode with 128 scans and 8 cm^−1^ resolution in a range of 2500–500 cm^−1^ on diamond crystal, and the surface potential was studied using a zeta analyzer (Malvern ZetasizerNano ZS90, Netherland).

### ZnONP-bacteria interfacial potential measurement

The mother cultures of all bacteria were prepared by inoculating a single bacterial colony into nutrient broth followed by incubation at 37°C with constant shaking at 150 rpm. For surface potential measurement at zeta analyzer, bacterial cells were harvested by centrifugation at 5000 rpm for 10 minutes at 4°C from the overnight culture, followed by two times washing using 1X phosphate buffer saline (PBS), and resuspended in PBS buffer prior to the measurement.

We followed the procedure adopted by Alves et al.^36^ for surface charge neutralization of *E. coli* by cationic antimicrobial peptide. However, in our study, ZnONP is used instead of cationic antimicrobial peptide. Briefly, 100 μL of bacteria culture in Muller Hinton Broth (MHB), grown overnight at 37°C and 150 rpm, was inoculated into 5 mL of fresh MHB. The culture was allowed to grow at 37°C until the bacterial concentration reaches ~3 × 10^8^ colony forming units/mL (optical density at 600 nm, O.D._600 nm_, ~0.1). The culture was diluted using fresh MHB to 3 × 10^5^ CFU/mL, followed by centrifugation at 13,000 rpm for 8 min, and the resulting pellet was washed two time using 10 mM HEPES buffer (pH 7.4) containing 150 mM NaCl. ZnONPs were suspended in same HEPES buffer with stock concentration of 5 mg/mL, and sonicated 5 min for proper dispersion. Dilutions of ZnONPs were prepared to final concentrations of 160, 250, 500, 1000, and 2500 μg/mL, using HEPES buffer. For neutralization reactions, 100 μL of the diluted ZnONPs were added to 900 μL of bacterial cells dispersed in HEPES buffer, and incubated for 1 hour at room temperature prior to zeta potential measurements. For positive control, bacterial cells were washed and dispersed in HEPES buffer to same dilution, and incubated for 1 hour at room temperature but without NP treatment. For zeta potential measurements and ROS study, we have taken *B. subtilis* and *E. coli* bacterium only, as representatives for Gram positive and Gram negative bacteria.

### Bacterial cell viability in presence of ZnONPs

The strain specific antibacterial activity of p-ZnONP were studied against the bacterium. All growth kinetic studies were performed by measuring O.D._600 nm_ using a UV-Vis spectrophotometer (Lambda 35, Singapore) with temperature controller peltier system (PTP 1+1 peltier system, Perkin Elmer, Singapore) at 37°C, in static condition. In an aseptic condition, 100 μL of respective bacteria culture was diluted to 3 mL using nutrient broth, followed by growth till mid log phase. Thereafter, an appropriate amount of p-ZnONP suspension (prepared in sterilized nutrient broth) was added to get the final p-ZnONP concentrations of 16, 25, 50, 100 and 250 μg/mL (additional 500 μg/mL data point was taken for *B. thuringiensis*) in culture, by keeping total volume constant. Culture without p-ZnONP was taken as positive control. In each case, p-ZnONP was added to the reaction mixture at mid log phase of growth kinetics, since at this phase the organisms are most potent/viable. Hence, the requirement of p-ZnONP to inhibit the growth is relatively high that lead to precise determination of the MIC of p-ZnONP. In addition to above study, similar growth kinetics of bacteria were performed for n-ZnONP.

We also used LIVE/DEAD BacLight Bacterial Viability Kit (L7007, Molecular probes, invitrogen) to distinguish viable and non-viable bacterial cells upon the NPs treatment, following the protocol suggested by the manufacturer using fluorescence microscope (Olympus IX71) with 20X objective lens.

The number of viable cells, after p-ZnONP treatment at mid log phase of their kinetics, were determined by CFU study. For the study, upon completion of the growth kinetics (from stationary phase) 10 μL of the bacterial samples were taken and spread on the nutrient agar plates after 10000 fold dilution. The plates were incubated overnight at 37°C. Colony forming units were quantified and compared with control to check the viability of bacterial cells upon treatment with varying concentrations of p-ZnONP.

### ROS detection

The production of ROS in the bacterial cultures treated with different concentrations of the ZnONPs was evaluated using DCFH-DA. *E. coli* and *B. subtilis* cultures were incubated at 37°C with 200 μM of DCFH-DA, and fluorescence emission was observed at 523 nm with excitation at 503 nm using micro-plate reader (Synergy H1 hybride reader, Biotek, USA). The stock concentration of DCFH-DA was calculated using 59,500 M^−1^cm^−1^ molar extinction coefficient at 500 nm. At the mid log phase of bacterial growth, the ZnONP suspensions were added to the final concentration of 16 and 250 μg/mL. To determine the ROS variation, the emission intensity of the treated cultures were compared with both, positive (without ZnONP treatment) and negative (culture media without DCFH-DA only) controls.

### Bacterial morphology upon ZnONP treatment

Initially, we visualized the morphology of bacteria upon p-ZnONP treatment using phase contrast microscopy (Olympus CKX41, JAPAN) with U-CMAD3 digital live camera and Q-capture pro7 software, taking samples directly upon the cover-slide from stationary phase of growth kinetics. To gain more insights into the morphological features, we scanned the samples using SEM and FE-SEM. For the imaging, samples were prepared using the protocol given by Jaysankar De et al., with some modifications^44^. In brief, from the stationary phase of growth kinetics, 1 mL of bacterial cultures were taken, and centrifuged at 5000 rpm for 5 mins at 4°C. The pellet was collected, washed twice, and resuspended in 1X PBS. One drop of the resuspended culture was put on glass slides, and bacterial cells were fixed by incubating overnight in 2.5% glutaraldehyde (prepared in 1X PBS). The fixed slides were suspended in 1% tannic acid for few minutes, and washed with distilled water followed by dehydration using increasing concentration of ethanol (30%, 50%, 70%, 90%, and 100%). The fixed, washed, and dehydrated bacterial cells were coated with platinum and gold for SEM (Jeol-JSM-6480 LV SEM, Japan) and FE-SEM (Nova NanoSEM 450/FEI) scanning, respectively.

## Author Contributions

M.A. contributed in preparing primary draft of the paper along with all the work carried out for the manuscript. While B.C.M. helped with his expert suggestions and comments on material chemistry, possibly involved, to rationalize the findings. M.S. helped with fluorescence microscopy experiments and image analysis. S.J. plotted the whole work and finalized the draft. All mentioned authors have reviewed the manuscript.

## Supplementary Material

Supplementary Information Supplementary Information

## Figures and Tables

**Table 1:** Minimum inhibitory concentration (MIC) of p-ZnONP towards different Gram positive and Gram negative bacteria

Bacteria Name	Gram positive/MIC	Gram negative/MIC
*Bacillus subtilis*	105. 17 ± 15.81	-
*Staphylococcus aureus*	118. 66 ± 21.66	-
*Bacillus thuringiensis*	120.27 ± 20.26	-
*Escherichia coli*	-	47.25 ± 9.29
*Shigella flexneri*	-	25.58 ± 5. 24
*Proteus vulgaris*	-	83.97 ± 6.7
