# Supplementary material for: The effects of interfacial potential on antimicrobial propensity of ZnO nanoparticle
Source: Sci Rep. 2015 Apr 15;5:9578. doi: 10.1038/srep09578 (PMC4397836; doi:10.1038/srep09578)
Supplement: Supplementary Information [file srep09578-s1.doc]

Supporting Information

The effects of interfacial potential on antimicrobial propensity of ZnO nanoparticle

Manoranjan Arakha^a^, Mohammed Saleem^a^, Bairagi C. Mallick^b^, Suman Jha^a*^

^a^Department of Life Science, National Institute of Technology Rourkela, Odisha 769008, India.

^b^Department of Chemistry, Ravenshaw University, Odisha 753003, India.


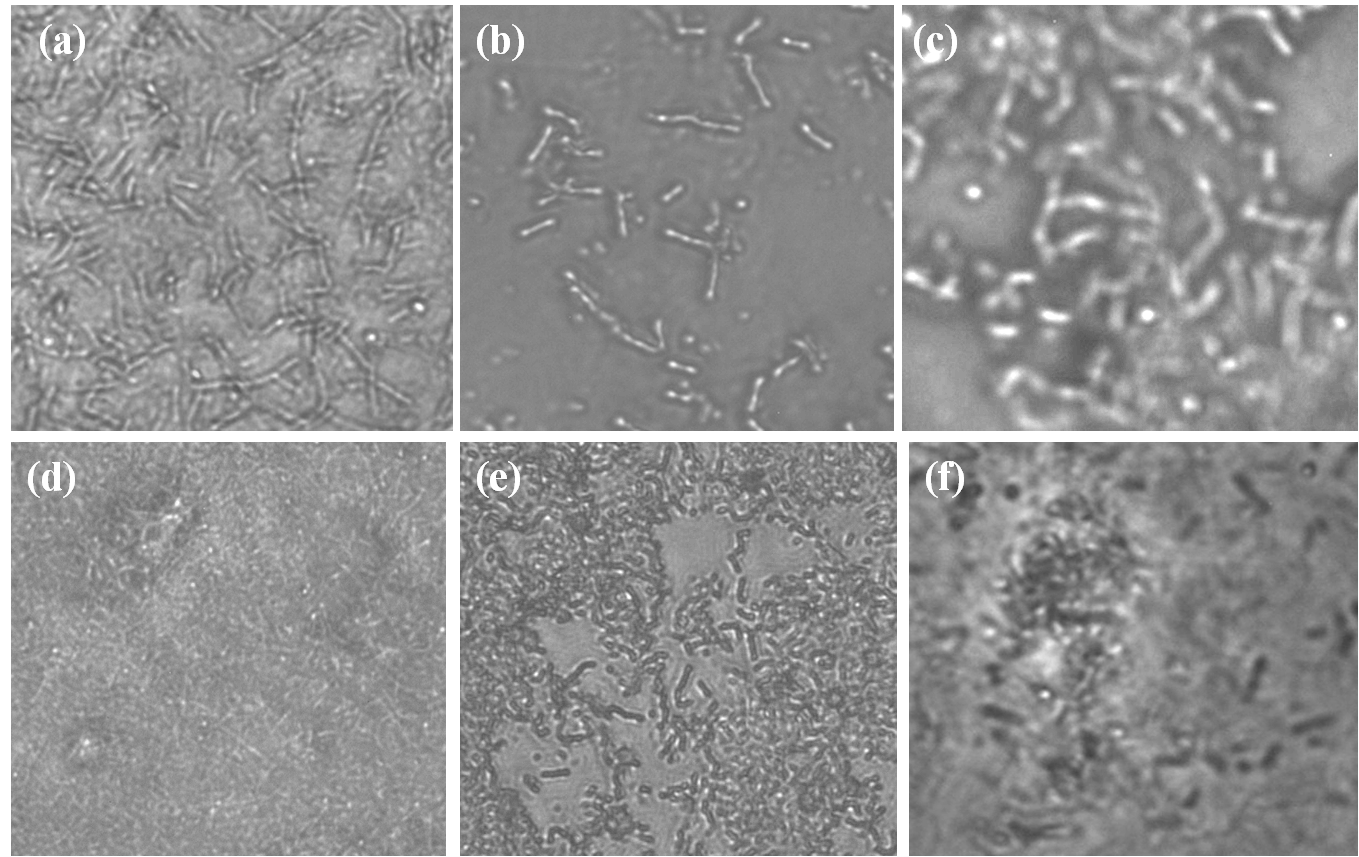


Figure S1 ǀ Morphological changes of Gram positive bacteria (*B. subtilis*, *B. thuringiensis* and *S. aureus*) at 100 µg/mL concentration of positively charged ZnO nanoparticle by phase contrast microscopy. Untreated cells of *B. subtilis* (a), *B. thuringiensis* (b), and *S. aureus* (c) show intact surface morphology, whereas ZnO nanoparticle treated cells show aggregation of cells (*B. subtilis* (d), *B. thuringiensis* (e), and *S. aureus* (f)), confirming bacterial cell membrane lysis.


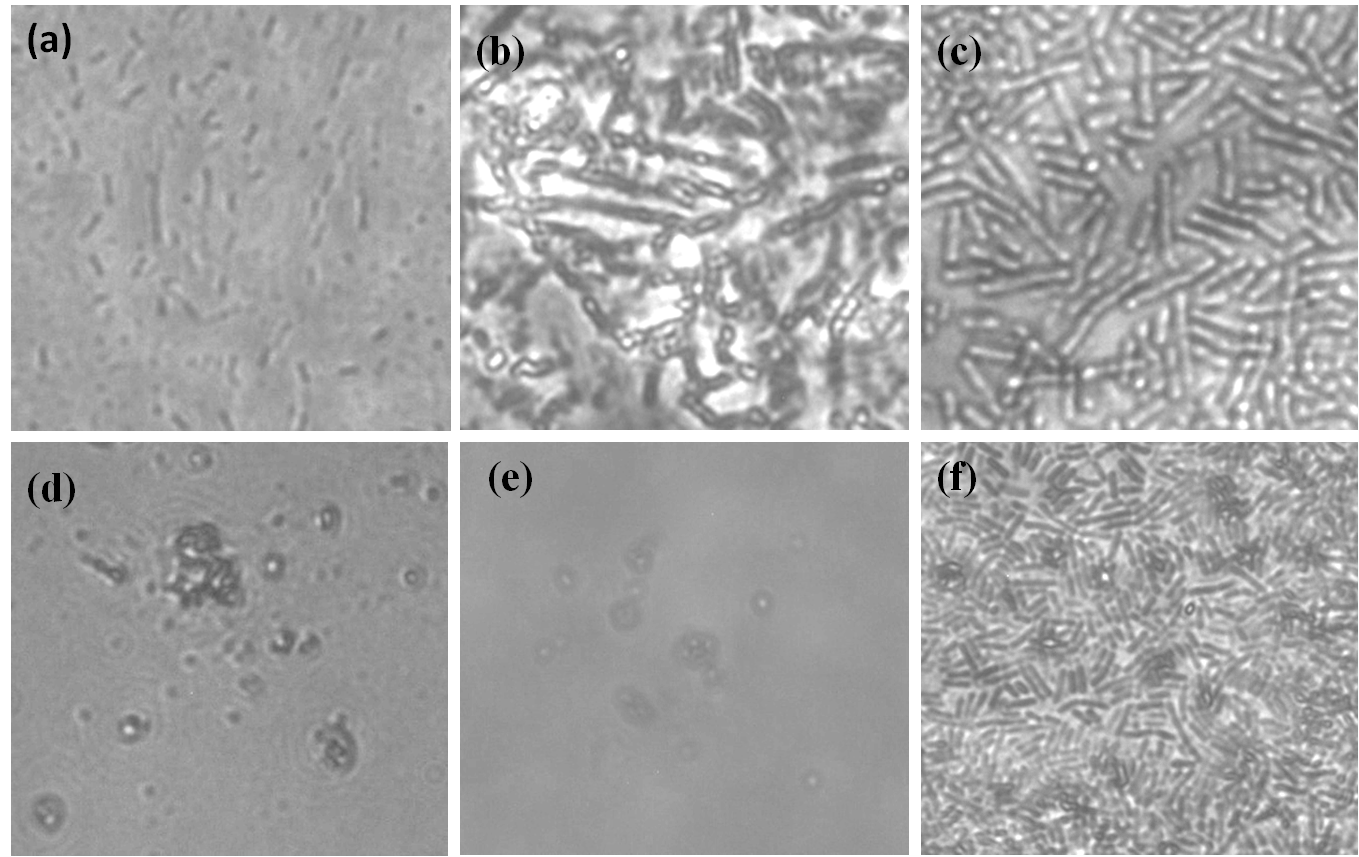


Figure S2 ǀ Morphological changes of Gram negative bacteria (*E. coli,* *S. flexneri* and *P. vulgaris*) at 50 µg/mL concentration of positively charged ZnO nanoparticle by phase contrast microscopy. Untreated cells of *E. coli* (a), *S. flexneri* (b), and *P. vulgaris* (c) show intact surface morphology, whereas ZnO nanoparticle treated cells show aggregation of cells (*E. coli* (d), *S. flexneri* (e), and *P. vulgaris* (f)) confirming bacterial cell membrane lysis.


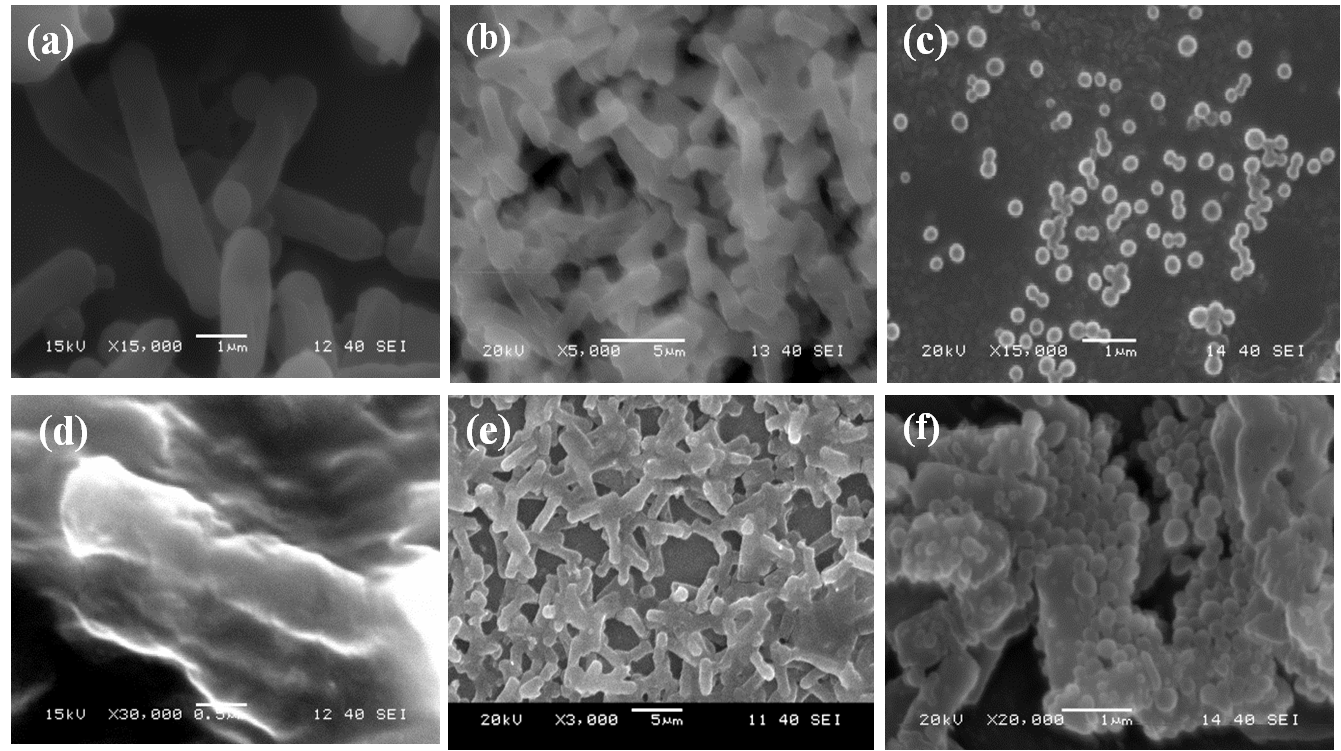


Figure S3 ǀ Morphological changes of Gram positive bacteria (*B. subtilis,* *B. thuringiensis* and *S. aureus*) at 100 µg/mL concentration of positively charged ZnO nanoparticle by SEM. Untreated cells of *B. subtilis* (a), *B. thuringiensis* (b), and *S. aureus* (c) show intact surface morphology, whereas ZnO nanoparticle treated cells show aggregation as well as membrane rupture of cells (*B. subtilis* (d), *B. thuringiensis* (e), and *S. aureus* (f)) confirming bacterial cell membrane lysis.


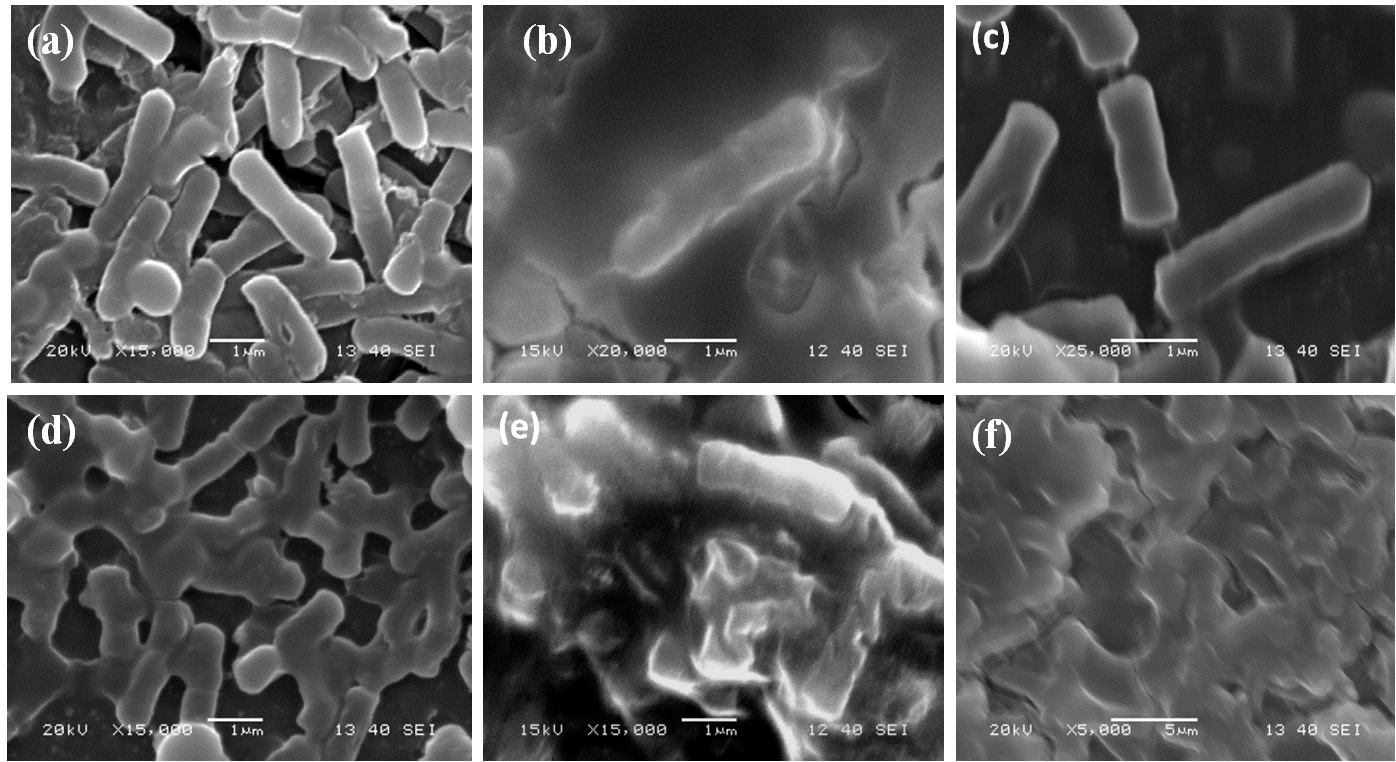


Figure S4 ǀ Morphological changes of Gram negative bacteria (*E. coli,* *S. flexneri* and *P. vulgaris*) at 50 µg/mL concentration of positively charged ZnO nanoparticle by SEM. Untreated cells of *E. coli* (a), *S.* *flexneri* (b), and *P. vulgaris*(c) show intact surface morphology, whereas ZnO nanoparticle at treated cells show aggregation as well as membranr rupture of cells (*E. coli* (d), *S.**flexneri* (e), and *P. vulgaris* (f)) confirming bacterial cell membrane lysis.


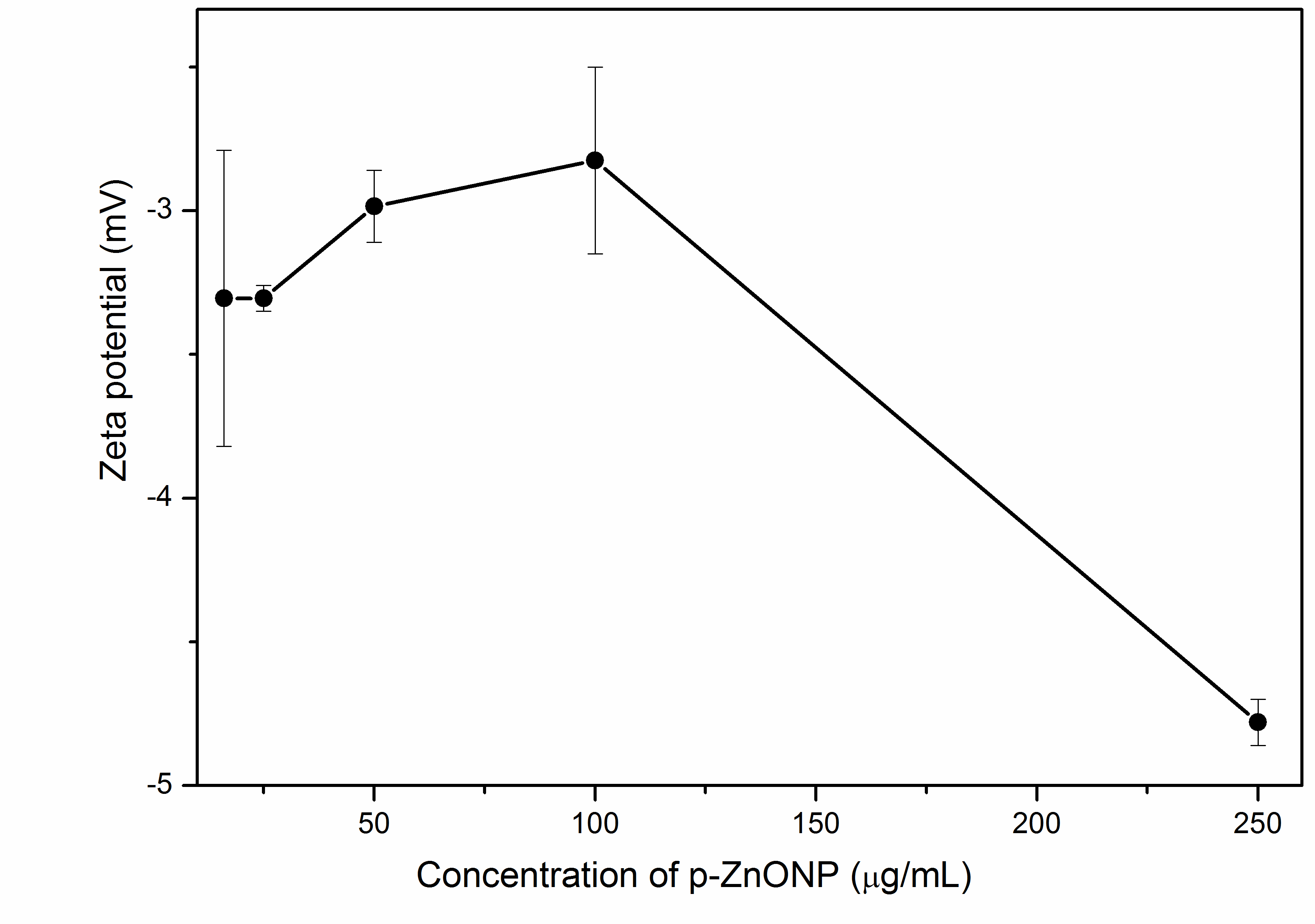


Figure S5 ǀ Zeta potential measurement of *B. subtilis* at different concentrations of p-ZnONP in Muller Hinton Broth (MHB). Unlike results obtained in Fig. 7 of the manuscript, which was performed in HEPES buffer, surface charge neutralization of *B. subtilis* is greatly affected in MHB, and this is due the presence of different biomolecular surfaces present in MHB during zeta potential measurement.
